# Verbal semantic expertise is associated with reduced functional connectivity between left and right anterior temporal lobes

**DOI:** 10.1093/cercor/bhae256

**Published:** 2024-06-19

**Authors:** Wei Wu, Paul Hoffman

**Affiliations:** School of Philosophy, Psychology & Language Sciences, University of Edinburgh, 7 George Square, Edinburgh EH8 9JZ, United Kingdom; Department of Music, Durham University, Palace Green, Durham DH1 3RL, United Kingdom; School of Philosophy, Psychology & Language Sciences, University of Edinburgh, 7 George Square, Edinburgh EH8 9JZ, United Kingdom

**Keywords:** semantic cognition, anterior temporal lobe, resting-state functional connectivity, knowledge representation

## Abstract

The left and right anterior temporal lobes (ATLs) encode semantic representations. They show graded hemispheric specialization in function, with the left ATL contributing preferentially to verbal semantic processing. We investigated the cognitive correlates of this organization, using resting-state functional connectivity as a measure of functional segregation between ATLs. We analyzed two independent resting-state fMRI datasets (*n* = 86 and *n* = 642) in which participants’ verbal semantic expertise was measured using vocabulary tests. In both datasets, people with more advanced verbal semantic knowledge showed weaker functional connectivity between left and right ventral ATLs. This effect was highly specific. It was not observed for within-hemisphere connections between semantic regions (ventral ATL and inferior frontal gyrus (IFG), though it was found for left–right IFG connectivity in one dataset). Effects were not found for tasks probing semantic control, nonsemantic cognition, or face recognition. Our results suggest that hemispheric specialization in the ATLs is not an innate property but rather emerges as people develop highly detailed verbal semantic representations. We speculate that this effect is a consequence of the left ATL’s greater connectivity with left-lateralized written word recognition regions, which causes it to preferentially represent meaning for advanced vocabulary acquired primarily through reading.

## Introduction

Our understanding of the world is shaped by our semantic knowledge (Jefferies 2013; Lambon Ralph et al. 2017). Convergent evidence from neuropsychology (Bozeat et al. 2000; Damasio et al. 2004; Butler et al. 2009), functional neuroimaging (Binder et al. 2011; Rice, Lambon Ralph, et al. 2015b; Visser and Lambon Ralph 2011) and brain stimulation studies (Pobric et al. 2007, 2010; Lambon Ralph et al. 2009) implicates both left and right anterior temporal lobes (ATLs) as critical regions for this semantic representation. One interpretation of these data could be that the semantic system shows redundancy, with both ATLs representing the same types of semantic information (Lambon Ralph et al. 2010; Snowden et al. 2012; Schapiro et al. 2013). However, there is limited empirical support for this *fully undifferentiated view*, since neuropsychological and neuroimaging studies suggest differential involvement of left and right ATL in different semantic tasks. Damage to left ATL has a greater impact on verbal semantic processing while right ATL damage can produce more significant deficits for faces and pictures (Gainotti 2007, 2013; Butler et al. 2009; Snowden et al. 2012; Rice et al. 2018). Left ATL has also been particularly implicated in naming and speech production (Damasio et al. 2004; Hoffman and Lambon Ralph 2018; Lambon Ralph et al. 2001; Rice, Lambon Ralph, et al. 2015b; Woollams et al. 2017), while right ATL may be more associated with social semantic processing (Olson et al. 2007; Zahn et al. 2007). These findings have led some researchers to propose a *fully specialized view* (Snowden et al. 2004; Gainotti 2012, 2014; Snowden et al. 2012) whereby left and right ATLs represent different forms of semantic knowledge associated with different modalities.

Between the extreme undifferentiated and fully specialized positions lies a *graded specialization view* (Guo et al. 2013; Hoffman and Lambon Ralph 2018; Rice, Hoffman, et al. 2015a; Rice, Lambon Ralph, et al. 2015b). Similar to the fully specialized view, the graded specialization view argues for differences in the function of left and right vATLs. However, the graded specialization model suggests that specialization is relative rather than absolute, with a relative left ATL bias for tasks requiring verbal knowledge or verbal output. On this view, the ATLs together act as an integrative “hub” for semantic knowledge representation (Hoffman and Lambon Ralph 2018; Lambon Ralph et al. 2010; Rice, Hoffman, et al. 2015a; Rice, Lambon Ralph, et al. 2015b). This graded specialization is similar to graded preferences for word recognition and face recognition in left and right ventral occipitotemporal cortex (VOTC) (Puce et al. 1996; Kanwisher et al. 1997; Cohen and Dehaene 2004; Thierry and Price 2006). A similar pattern of graded specialization has also been observed in inferior frontal gyrus (IFG), a key area for control and regulation of semantic processing (Thompson-Schill et al. 1997; Badre and Wagner 2007; Hoffman et al. 2010; Vitello and Rodd 2015). Parts of IFG show a left-hemisphere bias for verbal semantic tasks and a right-hemisphere bias for nonverbal tasks (Krieger-Redwood et al. 2015). Thus, graded hemispheric specialization appears to be a feature of semantic processing across multiple brain regions.

Though there is now considerable evidence for graded hemispheric specialization across the ATLs, little attention has been paid to how to this specialization develops. In other neural systems, the development of specific cognitive abilities drives increasing functional specialization (Uddin et al. 2010; Guerra-Carrillo et al. 2014). In VOTC, for example, right-lateralized responses to faces emerge as children learn to read (Monzalvo et al. 2012; Dehaene et al. 2015) and the strength of this lateralization is correlated with reading competence (Dundas et al. 2013; Dehaene-Lambertz et al. 2018). One explanation for this phenomenon, supported by computational models, is that orthographic processing is tightly coupled with left-lateralized speech production systems that are anatomically connected with left but not right VOTC (Plaut and Behrmann 2011; Behrmann and Plaut 2020). This asymmetry causes neurons in left VOTC to specialize for word recognition and, consequently, more of the computational work for face processing is taken up by the right VOTC. The result is increasing hemispheric specialization within a generally bilateral object recognition system. A similar mechanism could be at play in the ATLs (Hoffman and Lambon Ralph 2018; Woollams and Patterson 2018). From childhood onwards, reading is a critical modality for acquiring new vocabulary and general knowledge (Krashen 1989; Stanovich and Cunningham 1993; Cunningham and Stanovich 1998; Sullivan and Brown 2015). The development of advanced verbal semantics is therefore closely linked with the left-lateralized process of written word recognition, and this connectivity bias could lead the left ATL to specialize for representing verbal semantics. Right ATL specialization for nonverbal aspects of knowledge might then be an emergent consequence.

In the present study, we used resting-state fMRI to test the hypothesis that greater verbal semantic expertise is associated with greater hemispheric specialization in the semantic system. Resting-state functional connectivity (RSFC) is commonly used to measure the development of functional neural networks. In childhood, increasing age is associated with greater segregation (i.e. weaker correlations) between distinct networks, suggesting increasing differentiation in function (Uddin et al. 2010; He et al. 2019). For example, functional connectivity between left and right VOTC is lower in adults than in children (He et al. 2015). In adulthood, the acquisition of new skills is also associated with changes in the resting-state connectivity patterns of relevant networks (Zhu et al. 2011; Guerra-Carrillo et al. 2014). Acquisition of advanced semantic knowledge might therefore drive functional specialization that can be observed through RSFC. There is already some evidence for this at a global level: Wang et al. (2021) found that young adults with greater crystalized intelligence (measured using vocabulary tasks) displayed greater segregation between large-scale brain networks. Here, we took a more targeted approach to test whether the same is true for core nodes of the semantic system in ATL and IFG.

Our hypothesis is that the development of verbal semantic expertise in adulthood is associated with increasing specialization in function between left and right ATLs. There are two ways that this functional specialization might manifest itself. First, older people might show greater segregation between left and right ATLs than young people because they have accumulated more verbal semantic knowledge during their lives (Park et al. 2002; Verhaeghen 2003; Grady 2012; Hoffman 2018, 2019; Wu and Hoffman 2022, 2023a). Second, independent of age, people with more extensive verbal knowledge (indexed by vocabulary tests) may show greater segregation between ATLs. We tested these possibilities in two independent datasets that include RSFC data from a range of age groups. We focused on connectivity between the ventral surfaces of the ATLs (vATLs) as this region shows the most consistent involvement in semantic processing across a range of categories and input modalities (Lambon Ralph et al. 2017).

We also investigated whether parallel effects occur in the IFGs. IFG is involved in regulation and control of semantic processing, rather than representation of knowledge per se (Jefferies 2013; Lambon Ralph et al. 2017). Less is known about how these functions are arranged across left and right IFGs but there is some evidence for graded specialization mirroring that seen in the ATLs (Krieger-Redwood et al. 2015). If the development of more specialized semantic control functions leads to functional segregation in this region, then we would expect greater ability in verbal semantic control to be associated with more between-IFG segregation. Effects of aging may differ here, however. Semantic control ability begins to deteriorate with age and older people appear to rely more on bilateral IFG activation to regulate their semantic processing (Hoffman 2018, 2019; Hoffman and Morcom 2018; Wu and Hoffman 2022). Thus, segregation between IFGs might decrease with age.

## Materials and methods

We report two parallel sets of analyses on different datasets. We first conducted analyses on resting-state fMRI data collected as part of a larger study of age-related effects on semantic cognition (Wu and Hoffman 2023a, 2023b). These data are publicly available (https://osf.io/zbxt4) and the resting-state fMRI data have not been reported previously. To replicate our findings, we then conducted validation analyses using data from the Cam-CAN project repository (available at http://www.mrc-cbu.cam.ac.uk/datasets/camcan) (Shafto et al. 2014; Taylor et al. 2017).

### Participants

Forty-five older adults and 45 young adults were recruited from the University of Edinburgh Psychology department’s volunteer panel and local advertising and participated in the study in exchange for payment. All participants were native English speakers and reported to be in good health with no history of neurologic or psychiatric illness. The older participants completed the Mini-Addenbrooke’s Cognitive Examination (Hsieh et al. 2015) as a general cognitive screen. We excluded data from two older participants, who scored <26 of 30 on the Mini-Addenbrooke’s Cognitive Examination. Two young participants’ data were also excluded because of technical issues or structural abnormalities. Thus, data from 43 older participants (28 females, 15 males, mean age = 68.14 years, SD = 5.21 years, range = 60–79) and 43 young participants (31 females, 12 males, mean age = 23.07 years, SD = 3.23 years, range = 18–32) were used in the analyses. Both age groups had a high level of formal education (older adults years of education: mean = 15.65 years, SD = 2.84 years, range = 10–22; young adults: mean = 17.07 years, SD = 2.53 years, range = 12–23), and young adults had completed more years of education than older adults (*t*_84_ = 2.44, two-tailed *P* < 0.05). Informed consent was obtained from all participants and the research was performed in accordance with all relevant guidelines and regulations. The study was approved by the University of Edinburgh Psychology Research Ethics Committee. Sample size was determined by the resources available to complete the study.

*Validation analyses:* We used data from the Cam-CAN dataset (Shafto et al. 2014; Taylor et al. 2017) to conduct validation analyses. In this cohort, we first removed data from seven participants who had missing performance scores in more than eight behavioral tests across the two stages of the Cam-CAN project. This was because a large proportion of missing behavioral data may indicate the participant had abnormal cognitive ability. Then we focused on individuals whose fMRI data from resting-state and movie watching scans were both available. Data from 642 participants (324 females, 318 males, mean age = 54.65 years, SD = 18.46 years, range = 18.50–88.92) were used for analysis.

### Tasks

**Fig. 1:**
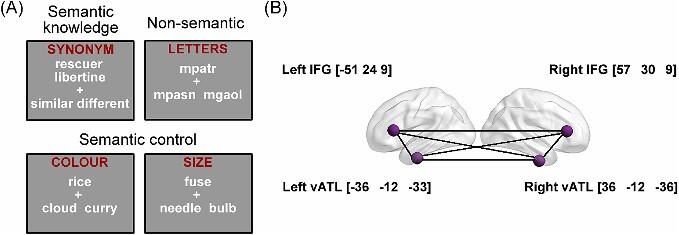
(A) Example items from each task in our dataset and (B) seed ROIs of left and right vATLs and IFGs and their MNI coordinates.

In our study, participants completed a verbal synonym judgment task, a verbal feature matching task and a cognitively demanding nonsemantic task during fMRI. Neuroimaging data for these tasks have been reported elsewhere (Wu and Hoffman 2023a, 2023b) and were not used for formal analyses (except for regions of interest [ROIs] definition) in the current study. Here, we used participants’ behavioral responses during scanning as the cognitive performance measures for the current study. The stimuli for all three tests were taken from the norms of Wu and Hoffman (2022) (see Fig. 1 for examples) and the tasks are similar to those used previously to measure semantic knowledge vs. control (Hoffman 2018, 2019).

*Test of verbal semantic knowledge*. We used an 80-item synonym-judgment task to probe participants’ verbal semantic knowledge (i.e. vocabulary). On each trial, two words along with two options (i.e. similar or different) were shown to the participant around the center of the screen. Participants were asked to decide if the two words shared a similar meaning or not. In line with the approach taken in vocabulary tests in standardized cognitive batteries (Wechsler 1958, 1981; Raven et al. 1989; Baddeley et al. 1992), words were taken from a wide range of word frequency, including a high proportion of low-frequency words whose meanings are less familiar to people. Thus it measured the breadth of verbal semantic knowledge that participants possessed.

*Test of verbal semantic control*. An 80-item feature-matching task was designed to probe participants’ ability to exercise cognitive control over the retrieval and selection of semantic knowledge. On each trial, participants were presented with a probe word along with two option words. They were asked to select the option that shared a particular semantic feature with the probe (40 color trials and 40 size trials). For instance, on a color trial, *sunflower* would match with *lemon* as both are typically yellow. This task demands semantic control processes as participants need to select the target semantic feature and inhibit competing but irrelevant semantic information from the distractor (e.g. *sunflower*—*stalk*) (Thompson-Schill et al. 1997; Badre et al. 2005). This was a verbal task as all stimuli were written words. However, because the features we used are related to visual properties of objects, it also required access to nonverbal aspects of semantic knowledge.

*Test of nonsemantic cognitive control*. We used an 80-item string-matching task to examine participants’ general executive control ability. Each trial in this task consisted of three meaningless letter strings (e.g. *mpatr*), and participants were asked to choose which of the two options had greatest orthographic similarity with the probe (i.e. sharing the most letters in the same order). This task examined general cognitive control processes, as participants needed to resolve competition between options without engaging semantic processing. We included this measure in analyses to control for nonspecific effects of cognitive performance.

*Validation analyses:* Performance data from two cognitive tests in the Cam-CAN battery were used: the Spot The Word test (STW, Baddeley et al. 1993) and a familiar face recognition test (Bartlett and Leslie 1986). The STW test requires a participant to identify the real words out of pairs of items comprising one word and one nonword. This test was conducted in stage 1 of the Cam-CAN project as a measure of verbal intelligence (Taylor et al. 2017), and it is comparable to the semantic knowledge task in our dataset as both examined breadth of verbal knowledge (i.e. people with more advanced verbal knowledge should correctly recognize more words). Scores on the STW test are strongly correlated with synonym judgment tasks (*r* > 0.8) (Hoffman 2018). We included the familiar face recognition test as a control. In this test, participants were shown 40 images of faces (30 famous and 10 unfamiliar) and asked to judge whether the face is familiar or not. For a face identified as familiar, participants were also asked to verbally provide the person’s name, and other information (e.g. occupation). The familiar face recognition task is a multimodal semantic test as it entails the linking of nonverbal (i.e. the face) with verbal semantic knowledge (i.e. the name). As such, we would not expect greater segregation between left and right vATLs to be associated with performance.

### Design and procedure

In our dataset, there were two task-fMRI scanning runs, in which participants completed the synonym judgment, feature matching and nonsemantic tasks in a blocked fashion. In each run, participants viewed 10 blocks from each of the three tasks. Each block started with a 2-second task cue (i.e. “synonym,” “color”/“size,” or “letters”), which remained at the top of the screen during the whole block (see Fig. 1 for example). After that, participants were presented with four 5-second task trials. The intertrial interval was 1–3 seconds (jittered, 2 seconds on average). The blocks were separated by 8-second fixations. Block order in each run was randomized. The trial order within each block was pseudorandomized to make sure that the position of correct responses in the block (i.e. left or right) was balanced. To counterbalance the potential influences of block and trial orders on performance, two sets of experimental programs with different block and trial orders were made and each set was used for half of the participants in each age group. Behavioral data from these two runs were used as performance measures in the current study. The neuroimaging data from these two runs were used to define the seed regions for resting-state analyses (see below). In between the two task-fMRI runs, resting-state fMRI data were acquired while participants rested with their eyes open. There was a fixation cross on the screen during the scan and this run lasted 6 minutes.

*Validation analyses:* We used data from two fMRI scans in the Cam-CAN data, including a resting-state scan which lasted for 8 min and 40 s, during which participants rested with their eyes closed and a movie-watching scan, in which participants passively viewed an 8-min excerpt of a film: Alfred Hitchcock’s “Bang! You’re Dead”. We included the movie data because the resting-state scan used a single-echo imaging sequence that is relatively insensitive to activation in the vATLs (Ojemann et al. 1997; Visser et al. 2010). In contrast, the movie scan used a multiecho sequence that has been shown to improve signal quality in this brain region (see next section). By combining data from both scans, we therefore improved sensitivity to effects in vATLs. However, qualitatively similar results were obtained when analyzing only the resting-state scan.

### Image acquisition and processing

For our dataset, images were acquired on a 3T Siemens Skyra scanner with a 32-channel head coil. fMRI in the vATL region is affected by susceptibility artifacts that can negatively impact data quality (Devlin et al. 2000). To combat this, we adopted a whole-brain multiecho acquisition protocol shown to improve ventral temporal fMRI signal as well as minimizing the effects of head movement (Halai et al. 2015; Kundu et al. 2017). The fMRI data were simultaneously acquired at three echo times (TEs), and then were weighted and combined and the resulting time series were denoised using independent component analysis (ICA). The multiecho Echo-Planar Imaging (EPI) sequence included 46 slices covering the whole brain with TE at 13 ms, 31 ms, and 50 ms, repetition time (TR) = 1.7 s, flip angle = 73°, 80 × 80 matrix, reconstructed in-plane resolution = 3 mm × 3 mm, slice thickness = 3.0 mm, and multiband factor = 2. One run of 212 volumes was acquired. For each participant, a high-resolution T1-weighted structural image was also acquired using an MP-RAGE sequence with 1 mm isotropic voxels, TR = 2.5 s, TE = 4.4 ms.

Images were preprocessed and analyzed using SPM12, the TE-Dependent Analysis Toolbox (Tedana) (DuPre et al. 2021) and the DPABI Toolbox (Yan et al. 2016). The first four volumes of the time series were discarded. Estimates of head motion were obtained using the first BOLD echo series. Slice-timing correction was carried out and images were then realigned using the previously obtained motion estimates. Tedana was used to combine the three echo series into a single-time series and to divide the data into components classified as either BOLD-signal or noise-related based on their patterns of signal decay over increasing TEs (Kundu et al. 2017). Components classified as noise were discarded. After that, images were unwarped with a B0 fieldmap to correct for irregularities in the scanner’s magnetic field. Functional images were spatially normalized to MNI space using SPM’s DARTEL tool (Ashburner 2007). To improve the quality of the resting-state fMRI signals, additional preprocessing procedures were conducted via DPABI (https://rfmri.org/DPABI, Yan et al. 2016), including: (i) linear detrending, (ii) regression of motion parameters (Friston-24 parameters) (Friston et al. 1996), mean white matter and cerebrospinal fluid signals, global signal, as well as outlier scans which had a framewise displacement (FD) larger than 0.3, (iii) temporal band-pass (0.01–0.1 Hz) filtering, and (iv) spatial smoothing with a Gaussian kernel of 6 mm full width half maximum. We chose to remove the whole-brain signal in the second step to reduce the effect of physiological artifacts (Fox et al. 2005; Birn et al. 2006; Yan and Zang 2010).

*Validation analyses:* We used preprocessed fMRI images from the Cam-CAN dataset. The resting-state fMRI data had 261 volumes and acquired with 32 slices with 3.7 mm slice thickness, TR = 1.97 s, TE = 30 ms, flip angle = 78°, FOV = 192 mm × 192 mm, resolution = 3 mm × 3 mm × 4.44 mm. As described by Taylor et al. (2017), preprocessing involved unwarping, realignment, slice-time correction and normalization. Data-driven wavelet despiking was applied to minimize motion artifacts (Patel et al. 2014). For the movie-watching data, there were 193 volumes scanned with a multiecho EPI sequence, TR = 2.47 s, TE = 9.4 ms, 21.2 ms, 33 ms, 45 ms and 57 ms, flip angle = 78°, FOV = 192 mm × 192 mm, resolution = 3 mm × 3 mm × 4.44 mm. The preprocessing procedures for the movie-watching data were similar to the resting-state data, except that ICA was performed to combine the five echo series. For both the resting-state and movie-watching data, we used DPABI to perform the same set of additional preprocessing procedures (with the same parameters) as for our dataset, including linear detrending, regression of nuisance variables, band-pass filtering and smoothing. Note that, because the Cam-CAN dataset had already undergone wavelet despiking processing, a new data-driven method that can be used to control head movement artifacts and replace traditional data scrubbing (Patel et al. 2014), we did not regress out outlier scans which had a large FD for the Cam-CAN dataset.

### Definition of regions of interest (ROIs)

To identify seed regions in left and right vATLs and IFGs, we used a method that combined anatomic masks with group-level task-activated peaks. Several steps were involved in this method.

First, we generated anatomical vATL and IFG masks. We defined the IFGs using the BA 45 mask in the Brodmann Areas Map provided with MRIcron (https://people.cas.sc.edu/rorden/mricro/template.html). We defined anatomical vATL regions in a similar fashion to Hoffman and Lambon Ralph (2018). That is, we generated an anatomical mask using the voxels with a greater than 50% probability of falling within fusiform gyrus in the LONI Probabilistic Brain Atlas (LPBA40), and we divided the fusiform gyrus mask into 5 roughly-equal-length sections that ran along an anterior-to-posterior axis. The anatomical masks of vATLs were then constructed by combining the anterior two sections of the above mask.

Second, using the two task-fMRI runs in our dataset (see Design and procedure section), we contrasted the average of the synonym judgment and feature matching tasks versus the nonsemantic task to identify voxels that were most responsive to semantic processing. We chose to combine the two semantic tasks instead of comparing each of them with the nonsemantic task separately, because our study aimed to define the core semantic regions (i.e. IFGs and ATLs) that are responsible for semantic processing in general but not limited to one specific task. By overlapping this functional map with the anatomical masks, we obtained group-level peak coordinates in the left and right vATLs and IFGs. Spheres with 8-mm radius were built centered on these coordinates, which were the final seed ROIs used in the functional connectivity analyses (shown in Fig. 1).

### Resting-state functional connectivity (RSFC) analyses

The same set of analyses were conducted for both our dataset and the Cam-CAN dataset. For each participant, timecourse in each seed ROI (i.e. left and right vATLs and IFGs) was extracted by averaging across all voxels in the ROI. Pearson correlations between timecourses were calculated for each pair of seeds. The resulting correlation coefficients were then Fisher-transformed to Z scores, which were used for group-level analyses. For the Cam-CAN data, this process was performed separately for the resting-state and movie-watching scans and the obtained Z scores were averaged for each participant to give a single Z score.

We first examined how intrinsic functional connectivity within the core semantic network (i.e. vATLs and IFGs) varied with age. For our dataset, which only included adults younger than 35 and older than 60, linear regression models were built to predict RSFC values of each pair of seed ROIs using age group as a categorical predictor. To account for potential effects of head motion on RSFC estimates, we also calculated the individual-level mean FD (mean FD) (Power et al. 2012) during the resting-state scanning and included it in each model as a covariate of no interest.

*Validation analyses:* For the Cam-CAN dataset, which includes participants across a wide range of ages, we treated age as a continuous variable and used it to predict the RSFC values of each pair of seed ROIs (i.e. the Fisher-z–transformed correlation values). The individual-level mean FD in the resting-state and movie-watching scans were averaged and included in the models as a covariate of no interest.

We next constructed a series of further linear regression models to investigate the relationship between RSFC and participants’ performance in the cognitive tasks. For each task in our dataset, we specified a regression model with age group, task performance (i.e. accuracy) and their interaction as predictors of RSFC values for each pair of seeds. Mean FD was included in the model as a covariate of no interest. This model was then compared with the previous model (i.e. the age effect model) to examine if the inclusion of task performance improved model performance in predicting RSFC values. These models revealed how task performance and its interaction with age were correlated with RSFC for each pair of ROIs. All continuous predictors were standardized prior to entry in our models. We used accuracy rather than reaction time as the behavioral measure because: (i) reaction times vary between age groups due to changes in general processing speed that are not specific to semantic cognition (Salthouse 1996), and (ii) we aimed to keep our behavioral measures consistent with the behavioral measures used in the Cam-CAN data. The distribution of task performance (i.e. accuracies in our dataset and performance scores in the Cam-CAN dataset) was shown in Supplementary Fig. S1.

*Validation analyses:* We used the total score in the STW task and performance in the face recognition task (i.e. number of faces correctly recognized as familiar subtracting false alarms) as the behavioral measures for the Cam-CAN dataset. For each task, we built a regression model with age and task performance as predictors to predict the RSFC values of each pair of seeds, and individual-level mean FD was included in the model as a covariate of no interest. This model was then compared with the corresponding age-only model. Age was treated as a continuous variable in the Cam-CAN models. All regression models were computed using the lme4 package in R (https://cran.r-project.org/) and p-values were corrected for multiple comparisons using the false discovery rate approach (Benjamini and Hochberg 1995).

It is worth noting that, although the RSFC of left–right IFGs and left–right vATLs were the main focus of the current study, we also investigated the other connections between IFGs and vATLs for completeness. We report the two key cross-hemispheric connections in the main paper and show the effects for other connections in Supplementary Information.

## Results

**Fig. 2:**
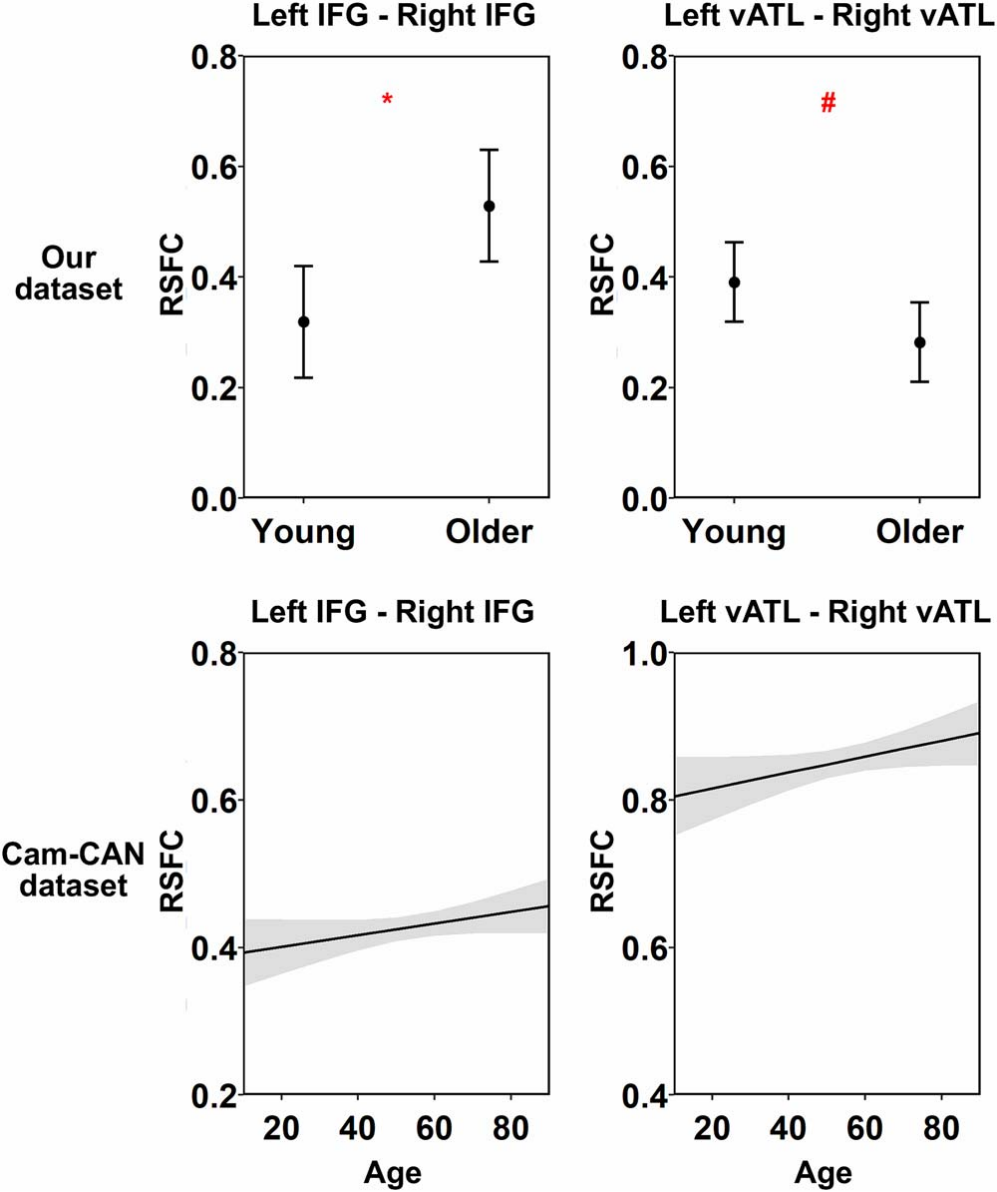
Results of linear regression models for age effects. This figure shows the modeled effects of age on RSFC between each pair of seed ROIs in each task. Shadow areas and error bars indicate 95% confidence intervals. The asterisks indicate significance level after FDR correction within dataset (i.e. 4 times for each dataset), # *P* = 0.070, * *P* < 0.05, ** *P* < 0.01, *** *P* < 0.001.

**Fig. 3:**
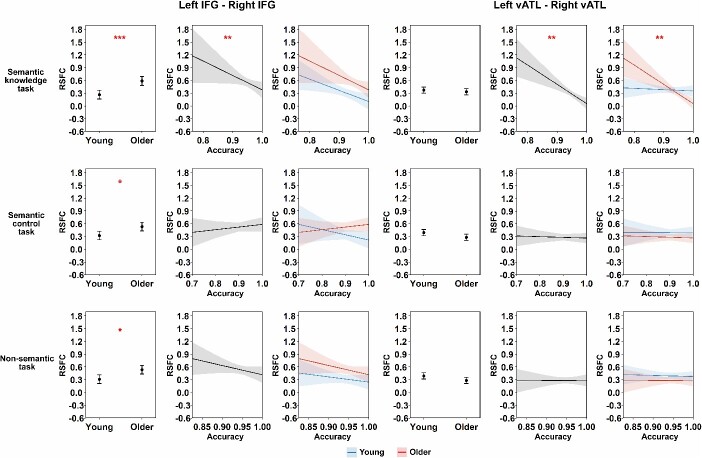
Results of linear regression models for our dataset. This figure shows the modeled effects of age group and task performance on RSFC between each pair of seed ROIs. The FD covariate effects were not shown for simplicity. Shadow areas and error bars indicate 95% confidence intervals. The asterisks indicate significance level after FDR correction within each task (i.e. 8 times for each task), * *P* < 0.05, ** *P* < 0.01, *** *P* < 0.001.

Using our dataset, we began by testing how connectivity between the left and right vATLs and IFGs varied across age groups. When controlling for FD, older people showed significantly stronger correlations between IFGs than young people (see Table 1 and Fig. 2). They showed weaker correlations between vATLs than young people but this effect did not survive correction for multiple comparisons (*P* = 0.07). Thus there was little support for the idea that functional specialization in the vATLs increases with age. Other connections did not vary between age groups (see Supplementary Table S1 and Fig. S2). We then tested whether cross-hemispheric RSFC was related to performance in semantic and nonsemantic tasks. We did this by adding performance on each of the three tasks (and their interaction with age) separately to the model in turn and testing whether this improved model fit. The addition of semantic knowledge scores improved the fit of the left vATL—right vATL (*F* = 7.53, *P* < 0.01) and left IFG—right IFG (*F* = 4.73, *P* < 0.05) models. As shown in Table 2 and Fig. 3, people with better performance on the synonym judgment task exhibited weaker left vATL—right vATL connectivity (performance effect: *B* = −0.094, *SE* = 0.028, *P* < 0.01) and weaker left IFG—right IFG connectivity (performance effect: *B* = −0.117, *SE* = 0.040, *P* < 0.01). Thus, people with greater verbal semantic expertise appeared to show more functional specialization between left and right semantic regions. For the vATLs, this was qualified by an age × performance interaction, as the semantic knowledge effect was stronger in older people. Effects of age remained significant for IFGs but not vATLs (see Table 2), suggesting that the weak effect of lower bilateral vATL connectivity in later life could be due to development of more advanced verbal semantic knowledge.

**Table 1:** Results for age effects in the intrinsic functional connectivity analysis in two datasets.

		Left IFG—Right IFG			Left vATL—Right vATL		
	Effect	*B*	*SE*	*p*	*B*	*SE*	*p*
Our dataset	Age group	0.105	0.039	**<0.05**	−0.054	0.028	0.070
	FD	−0.107	0.039	**<0.05**	−0.001	0.028	0.980
Cam-CAN dataset	Age	0.015	0.009	0.144	0.020	0.011	0.120
	Averaged FD	0.005	0.009	0.560	0.039	0.011	**<0.01**

Note: Results of linear regression analysis are presented. The *p* values are FDR corrected for each dataset separately (i.e. 4 times for each dataset). Significant *p* values are highlighted in bold. FD = framewise displacement.

**Table 2:** Results for linear regression testing the relationship between RSFC and expertise in our dataset.

		Left IFG—Right IFG			Left vATL—Right vATL		
	Effect	*B*	*SE*	*p*	*B*	*SE*	*p*
Semantic knowledge task	Age group	0.161	0.041	**<0.001**	−0.019	0.029	0.572
	Performance	−0.117	0.040	**<0.01**	−0.094	0.028	**<0.01**
	FD	−0.163	0.042	**<0.001**	−0.027	0.029	0.466
	Age group × Performance	−0.015	0.037	0.698	−0.082	0.026	**<0.01**
Semantic control task	Age group	0.105	0.039	**<0.05**	−0.054	0.028	0.147
	Performance	−0.016	0.035	0.968	−0.005	0.025	0.968
	FD	−0.109	0.039	**<0.05**	−0.001	0.028	0.968
	Age group × Performance	0.050	0.035	0.303	−0.004	0.025	0.968
Non-semantic task	Age group	0.112	0.039	**<0.05**	−0.053	0.028	0.166
	Performance	−0.058	0.034	0.193	−0.003	0.025	0.927
	FD	−0.121	0.040	**<0.05**	−0.003	0.029	0.927
	Age group × Performance	−0.016	0.034	0.927	0.006	0.024	0.927

Note: The *p* values are FDR corrected for each task separately (i.e. 8 times for each task). Significant *p* values are highlighted in bold. FD = framewise displacement.

Semantic knowledge did not predict the strength of other IFG-vATL connections (i.e. adding these predictors did not improve model fit, *F*s < = 0.49, *ps* > = 0.61; see Supplementary Table S2 and Fig. S3 for results of these models). Feature-matching task performance and nonsemantic task performance were not associated with connection strength for any connections, indicating that effects were specific to the verbal knowledge representation and not to other aspects of semantic cognition or general cognitive ability. The absence of meaningful effects for the other tasks was verified using the Two One-Sided Tests (TOST) approach (Lakens 2017). The TOST can be used to specify a lower and upper effect-size bound based on sample size and test if a given effect falls within this range. A significant result indicates the absence of an effect that is worthwhile to examine under the defined threshold. In our study, for the correlation between left–right vATLs RSFC and feature-matching task performance, the TOST procedure indicated that the observed effect size (*r* = −0.01) was significantly within the equivalent bounds of *r* = −0.31 and *r* = 0.31 (i.e. a medium effect), *P* = 0.002. This means that we can reject the null hypothesis that |*r*| > 0.31. For the nonsemantic task, the TOST procedure also indicated that the observed effect size (*r* = −0.02) was significantly within the equivalent bounds of *r* = −0.31 and *r* = 0.31, *P* = 0.003.

**Fig. 4:**
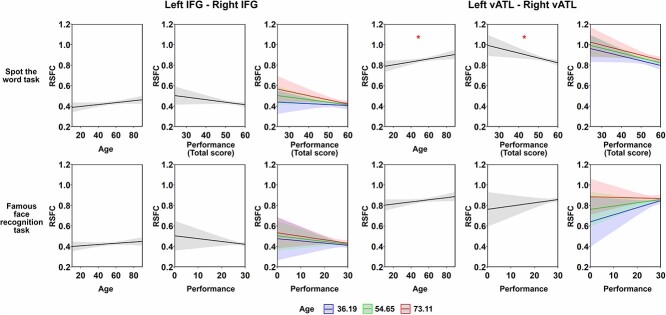
Results of linear regression model analysis for Cam-CAN dataset. This figure shows the modeled effects of age and task performance on RSFC between each pair of seed ROIs. The FD covariate effects were not shown for simplicity. Age × performance interaction is illustrated by plotting performance effects at the mean age and plus/minus 1 SD. Shadow areas and error bars indicate 95% confidence intervals. The asterisks indicate significance level after FDR correction within each task (i.e. 8 times for each task), * *P* < 0.05, ** *P* < 0.01, *** *P* < 0.001.

*Validation analyses:* For the Cam-CAN dataset, we found no age effects on RSFC for left IFG—right IFG or left vATL—right vATL (Table 1 and Fig. 2). In contrast, other IFG-vATL connections exhibited a significant decrease in strength with age (Supplementary Table S1 and Fig. S2). For the task performance models, inclusion of STW scores improved the fit of the left vATL—right vATL model (*F* = 3.73, *P* < 0.05). As shown in Table 3 and Fig. 4, people with better performance on the STW task exhibited weaker left vATL—right vATL connectivity (performance effect: B = −0.026, SE = 0.009, *P* < 0.05), replicating the effect of synonym judgment task in our dataset. This effect was present across ages (i.e. it did not interact with age). Unexpectedly, however, when controlling for STW performance, a positive effect of age emerged: connectivity between vATLs increased with age.

**Table 3:** Results for linear regression testing the relationship between RSFC and expertise in the Cam-CAN dataset.

		Left IFG—Right IFG			Left vATL—Right vATL		
	Effect	*B*	*SE*	*p*	*B*	*SE*	*p*
Spot the word task	Age	0.017	0.009	0.129	0.027	0.011	**<0.05**
	Performance	−0.013	0.008	0.156	−0.026	0.009	**<0.05**
	Averaged FD	0.004	0.009	0.776	0.035	0.011	**<0.01**
	Age × Performance	−0.008	0.007	0.345	−0.001	0.009	0.928
Famous face recognition task	Age	0.011	0.009	0.399	0.019	0.011	0.327
	Performance	−0.009	0.008	0.399	0.010	0.010	0.399
	Averaged FD	0.006	0.009	0.611	0.039	0.011	**<0.01**
	Age × Performance	−0.002	0.007	0.760	−0.012	0.008	0.335

Note: The *p* values are FDR corrected for each task separately (i.e. 8 times for each task). Significant *p* values are highlighted in bold. FD = framewise displacement.

Inclusion of STW scores did not improve the fit of the left IFG—right IFG model (*F* = 1.99, *P* = 0.14). This result diverges from our dataset, where we found that people with greater verbal semantic knowledge showed lower left IFG—right IFG connectivity. Here, the effect was in the same direction but was not statistically significant (two-tailed corrected *P* = 0.156). Inclusion of famous face performance did not improve fit of either left IFG—right IFG or left vATL—right vATL models (both *F* < = 1.39, both *p* > = 0.25), indicating that the observed effects in vATLs were specific to verbal semantic knowledge. The absence of meaningful effect was verified using the TOST approach (Lakens 2017), that is, for the correlation between left–right vATLs RSFC and famous face task performance, the TOST procedure indicated that the observed effect size (*r* = −0.02) was significantly within the equivalent bounds of *r* = −0.12 and *r* = 0.12 (i.e. a small effect), *P* = 0.005. For the other connections (see Supplementary Table S3 and Fig. S4), adding face recognition performance did improve model fit for the left IFG—right vATL RSFC (*F* = 3.64, *P* < 0.05). Better face recognition performance was linked with stronger correlation between these two regions (performance effect: B = 0.015, SE = 0.006, *P* < 0.05). STW performance did not predict the strength of any other connections.

## Discussion

The semantic network exhibits graded hemispheric specialization, with verbal semantic processing more closely associated with left-hemisphere regions (Gainotti 2014; Rice, Hoffman, et al. 2015a; Rice, Lambon Ralph, et al. 2015b; Snowden et al. 2012). We investigated the cognitive correlates of this specialization using two independent resting-state fMRI datasets. Connectivity between left and right vATLs was lower in people with more advanced verbal semantic expertise, as indexed by tasks that probed their ability to recognize and understand low-frequency words. This effect was first found in our study of 86 young and older adults (Wu and Hoffman 2023a) and then replicated in the Cam-CAN dataset (Shafto et al. 2014; Taylor et al. 2017), which includes over 600 participants across a wide range of ages. The effect was highly specific. Connectivity between vATLs was not correlated with performance on tasks that probed semantic control ability, nonsemantic processing or face recognition. And while verbal semantic expertise was correlated with connectivity between left and right vATLs (and in the Wu & Hoffman data, between left and right IFGs), it was not correlated with the strength of within-hemisphere connections between semantic regions. Thus, there appears to be a specific and replicable relationship between the amount of verbal semantic knowledge a person has and the degree to which the left and right-hemisphere elements of their semantic system are functionally segregated from one another. This new insight is important for understanding the underlying causes of hemispheric specialization in the semantic network.

Our interpretation of these findings is similar to that proposed by Behrmann, Plaut and colleagues for graded hemispheric specialization in VOTC (Plaut and Behrmann 2011; Dundas et al. 2013, 2015; Behrmann and Plaut 2014; Behrmann and Plaut 2020). It follows theories of graded semantic representation in the brain (Lambon Ralph et al. 2017; Plaut 2002; Rice, Hoffman, et al. 2015a) and involves three key assumptions. First, in line with the hub-and-spoke model (Lambon Ralph et al. 2017), we assume that the core computational function of the ATLs is to integrate inputs from a range of modality-specific processing streams. The ATLs generate semantic representations by extracting statistical regularities from these inputs. Second, we assume that the inputs received by different parts of the ATLs vary as a function of their connectivity with other neural systems. This variation occurs *within* each ATL, for example, the anterior portions of the superior middle temporal gyri are more strongly connected with auditory processing streams, while the anterior fusiform and parahippocampal gyri show stronger connections with VOTC (Binney et al. 2012). But more pertinently, we assume that it also occurs *across* hemispheres. Intra-hemispheric connections are much more abundant than cross-hemispheric connections, resulting in a stronger connection between the left ATL and left-lateralized orthographic processing and language production regions. The final assumption is that specialization in knowledge representation is determined by these variations in connectivity: neurons participate most strongly in representing concepts which are prominently featured in the inputs they receive. This specialization is graded rather than absolute because representations are highly distributed across the entire ATL system (Schapiro et al. 2013).

Our results follow naturally from this account. The tests we used to measure verbal semantic expertise probe knowledge of low-frequency words that are typically acquired late in development, during adolescence and adulthood. As this knowledge is primarily acquired through exposure to written language (Krashen 1989; Stanovich and Cunningham 1993; Cunningham and Stanovich 1998; Sullivan and Brown 2015) and written word recognition is left-lateralized (Dehaene et al. 2015), we suggest that the left ATL is more able to represent this knowledge than the right. Thus, as people develop more sophisticated and diverse verbal semantic representations, left ATL regions become relatively specialized for this aspect of semantic processing, driving increased functional differentiation between left and right ATLs. This differentiation can be observed in terms of lower levels of resting-state connectivity between left and right ATLs in people whose verbal semantic representations are more developed.

Our preferred account emphasizes *written* word processing as a key driver of ATL specialization. This claim is consistent with evidence that the left ATL bias for verbal semantics is strongest for written words and less prominent for spoken word processing (Hoffman and Lambon Ralph 2018; Marinkovic et al. 2003; Rice, Lambon Ralph, et al. 2015; Spitsyna et al. 2006). It also aligns with another resting-state fMRI study which found that people with more verbal semantic expertise (measured using a written synonym judgment task) showed stronger connections between left ATL and orthographic cortex in left VOTC (Mollo et al. 2016). However, it is important to note that we do have direct evidence for this claim, as we did not measure reading habits in our participants. Future studies could test the proposed link between left ATL specialization and written word processing by, for example, comparing functional connectivity in literate vs. illiterate adults (e.g. Resende et al. 2023) or by surveying participants on frequency of reading different types of material (e.g. Sullivan and Brown 2015). There are also other lateralization biases that explain hemispheric specializations in the ATLs. Left ATL is more connected with left-lateralized speech production systems than right ATL, and this connectivity bias might be a driver of specialization for verbal semantic processing (Lambon Ralph et al. 2001; Schapiro et al. 2013). Thus, while our data show that greater verbal semantic expertise is associated with greater between-ATL specialization, full understanding of the root causes of this effect will require further investigation.

If left ATL regions come to preferentially represent verbal semantic knowledge, are there other forms of semantic processing that rely preferentially on the right ATL? To answer this question, we need to consider which aspects of semantic representation rely on right-lateralized processing streams. Semantic representations for people is one candidate, given that face recognition processing in VOTC is right-lateralized (Plaut and Behrmann 2011; Behrmann and Plaut 2020). Patients with right ATL damage show more severe familiar face recognition deficits than those with left ATL damage (Snowden et al. 2012; Rice et al. 2018). If face/person expertise is the right-hemisphere counterpart to verbal semantic expertise, then we might expect people with highly developed person knowledge to also show a high level of segregation between ATLs. We did not find this: in the Cam-CAN data, we found no relationship between inter-ATL connectivity and performance on a famous face recognition task. It is important to note, however, in this test, participants were asked name the face and to verbally provide their occupation and nationality. Although face processing may be right-lateralized, linking objects and people to their names is highly reliant on left ATL regions (Lambon Ralph et al. 2001; Damasio et al. 2004). Thus this task may have required a combination of representations with greater reliance on each ATL. Indeed, other than written words and faces, most meaningful stimuli seem to activate the ATLs in a relatively bilateral fashion (Hoffman and Lambon Ralph 2018; Rice, Hoffman, et al. 2015a) so we may not expect other knowledge types to correlate with functional segregation of the ATLs. For example, Alam et al. (2021) found that people who were highly proficient in identifying images of famous landmarks showed *higher* resting-state connectivity between left and right ATLs. Knowledge of landmarks is likely to be acquired using inputs from neural networks supporting visual scene processing and spatial navigation. These systems are not strongly lateralized (Epstein and Baker 2019) so there is no connectivity-based impetus for one ATL to specialize in representing this knowledge.

Although our main focus in this study was on left and right vATLs, we also tested whether resting-state connectivity between left and right IFGs correlates with semantic task performance. Here we found a more mixed picture. In the Wu and Hoffman (2023a) data, more advanced verbal semantic knowledge was associated with weaker inter-IFG connectivity, mirroring the result in the vATLs. In the Cam-CAN data, however, a smaller effect in the same direction was not statistically significant (two-tailed corrected *P* = 0.156). Further research is needed to establish the robustness of this effect. If, however, such an effect is found reliably, this may indicate the factors driving functional segregation in the ATLs have similar effects in the IFGs.

Functional connectivity did not correlate with performance on a feature-matching task designed to probe semantic control ability (Thompson-Schill et al. 1997; Badre et al. 2005). It is important to note that this task was not as exclusively verbal in nature as the semantic knowledge tasks. Although the stimuli were presented as written words, the semantic control task required participants to make judgments about specific object properties (color, size) that are typically experienced through vision rather than language. This kind of nonverbal visual knowledge is less likely to show laterality effects, as object recognition is not subject to the same left-hemisphere biases as word recognition. In addition, the feature-matching task was not designed to probe the depth of participants’ semantic knowledge but rather their ability to resolve competition between active semantic representations. This semantic control ability relies heavily on the IFGs (Thompson-Schill et al. 1997; Badre and Wagner 2007; Hoffman et al. 2010; Vitello and Rodd 2015). While IFG activation for verbal semantic control processes is strongly left-lateralized, we have argued that right IFG also contributes to these processes when task demands are particularly high (Wu and Hoffman 2023a). On this basis, one might expect people with poorer semantic control ability to show greater inter-IFG connectivity, as they rely more often on right IFG to support the functions of the left. This hypothesis would be best tested in future studies that manipulate the need for control in purely verbal semantic decisions (e.g. resolving competition between the meanings of ambiguous words).

Finally, we investigated how cross-hemispheric functional connectivity in the semantic system varies with age. In the Wu and Hoffman (2023a) data, correlations between left and right IFGs were higher in the older age group. This greater cross-hemispheric interaction could indicate that older people rely more on right IFG to support left IFG in regulating semantic activation (Hoffman and Morcom 2018). This would be consistent with models that predict shifts towards greater bilaterality in later life to compensate for declines in cognitive function (Cabeza 2002; Berlingeri et al. 2013). However, this effect was not replicated in the Cam-CAN data, which may indicate that it is not reliable or that it follows a nonlinear trajectory in midlife which our analysis was not sensitive to. For the vATLs, our dataset showed a weak (nonsignificant) decrease in connection strength in older people that did not persist once verbal semantic knowledge was controlled for. In the Cam-CAN data, connectivity actually increased with age, after controlling for verbal semantic expertise. Thus, while effects of semantic expertise were strong and consistent across datasets, the effects of age (after controlling for expertise) were weaker and inconsistent. These findings suggest that it is primarily the acquisition of specific verbal knowledge that leads to increasing specialization across ATLs, rather than simply the passage of time.

In the Wu and Hoffman (2023a) data, the relationship between verbal semantic expertise and ATL segregation was only found in the older age group. The lack of an effect in the young age group may be due to a lack of variability in semantic knowledge in this group. Our younger participants were mostly aged in their early twenties and were almost all university students. It’s likely that levels of semantic knowledge varied less in this homogeneous group than they did in our older group. In contrast, the Cam-CAN data includes participants from a wide range of life stages. In this data, no interaction between the effect of verbal expertise and age was found, suggesting that the relationship between verbal semantic expertise and ATL segregation is stable across adulthood.

In summary, this study has established a specific and replicable relationship between individuals’ level of verbal semantic knowledge and the degree of functional segregation between their left and right vATLs. These findings support graded specialization theories of ATL organization (Guo et al. 2013; Lambon Ralph et al. 2017; Rice, Hoffman, et al. 2015a; Rice, Lambon Ralph, et al. 2015b). They suggest that this organization is not innate but is rather an emergent consequence of developing expertise in verbal semantic knowledge. The underlying causes of this effect await confirmation in future work, though we propose that hemispheric biases in written word recognition processes are a likely driving factor.

## Open practices

The current study uses data collected by Wu and Hoffman (2023). The behavioral data (10.7488/ds/3845 and 10.7488/ds/3846), resting-state fMRI data and analysis code (https://osf.io/zbxt4) are publicly available. All task stimuli were obtained from the norms of Wu and Hoffman (2022), with digital study materials available at https://osf.io/9px7g.

## Supplementary Material

Semantic_age_RSFC_supplementary_info_submit_R1_final_bhae256
